# Cumplimiento del tratamiento farmacológico en enfermedades crónicas no transmisibles en la población colombiana: revisión sistemática y metaanálisis

**DOI:** 10.7705/biomedica.7077

**Published:** 2023-12-29

**Authors:** Catalina Cáceres, Álvaro José Lora, Silvia Juliana Villabona, María Catalina Rocha, Paul Anthony Camachoi,

**Affiliations:** 1 Unidad de Diseño y Desarrollo, Fundación Oftalmológica de Santander, Floridablanca, Colombia Fundación Oftalmológica de Santander Floridablanca Colombia; 2 Facultad de Ciencias de la Salud, Universidad Autónoma de Bucaramanga, Bucaramanga, Colombia Universidad Autónoma de Bucaramanga Facultad de Ciencias de la Salud Universidad Autónoma de Bucaramanga Bucaramanga Colombia

**Keywords:** enfermedad crónica, enfermedades no transmisibles, cumplimiento del tratamiento, hipertensión, diabetes mellitus, enfermedad pulmonar obstructiva crónica, dislipidemias, accidente cerebrovascular, asma, Chronic disease, noncommunicable diseases, treatment adherence and compliance, hypertension, diabetes mellitus, pulmonary disease, chronic obstructive, dyslipidemias, stroke, asthma

## Abstract

**Introducción.:**

Las enfermedades crónicas no transmisibles representan la principal causa de muerte en el mundo y su prevalencia va en aumento debido a la transición epidemiológica. A pesar de los avances en su manejo, las cifras de control son deficientes y esto se atribuye a múltiples factores, como el cumplimiento del tratamiento farmacológico, que es uno de los más representativos y menos estudiados en la población colombiana.

**Objetivo.:**

Establecer la frecuencia de casos que cumplieron con el tratamiento farmacológico en pacientes colombianos con hipertensión arterial, enfermedad cerebrovascular, diabetes mellitus, asma, enfermedad pulmonar obstructiva crónica y dislipidemia, entre el 2005 y el 2022.

**Materiales y métodos.:**

Se llevó a cabo una revisión sistemática de la literatura y un metaanálisis de los estudios identificados mediante las bases de datos Medline y LILACS para sintetizar cuantitativamente el porcentaje de cumplimiento del tratamiento.

**Resultados.:**

Catorce estudios cumplieron los criterios de inclusión y se analizaron 5.658 pacientes. El cumplimiento del tratamiento fue del 59 %, con una heterogeneidad alta entre los estudios incluidos (IC_95%_ = 46-71 %; I2 = 98,8 %, p<0,001). Se obtuvo un mayor cumplimiento para la diabetes mellitus" (79 %; IC_95%_ = 65-90 %) y la dislipidemia (70 %; IC_95%_ = 66-74 %). En los pacientes con hipertensión arterial el cumplimiento fue del 51 % (IC_95%_ = 31-72 %).

**Conclusiones.:**

La revisión sistemática muestra un bajo cumplimiento de las recomendaciones sobre el manejo farmacológico de enfermedades crónicas no transmisibles, lo que puede repercutir en los resultados clínicos y en la carga de la enfermedad a largo plazo.

## Abstract (en)

**Introduction.:**

Non-communicable chronic diseases represent the leading cause of death worldwide, and their prevalence is increasing due to the epidemiological transition. Despite the advances in their management, control rates are deficient, attributed to multiple factors like adherence to pharmacological treatment, one of the most significant and least studied in the Colombian population.

**Objective.:**

To calculate adherence to treatment in Colombian patients with arterial hypertension, cerebrovascular disease, diabetes mellitus, asthma, chronic obstructive pulmonary disease, and dyslipidemia between 2005 and 2022.

**Materials and methods.:**

We performed a systematic literature review and a meta-analysis of studies identified through the Medline and LILACS databases to quantitatively synthesize treatment adherence percentage.

**Results.:**

Fourteen studies met the inclusion criteria, and 5,658 patients were analyzed. The treatment adherence was 59%, with significant heterogeneity among the included studies (95% CI= 46- 71%; I2 = 98.8%, p< 0.001). Higher adherence rates were observed for diabetes mellitus (79%; 95% CI = 65- 90%) and dyslipidemia (70%; 95% CI = 66- 74%). Adherence to arterial hypertension treatment was 51% (95 %; CI = 31- 72%).

**Conclusions.:**

This systematic review showed low adherence to recommendations regarding pharmacological management in non-communicable chronic diseases, which can have implications for long-term clinical outcomes and disease burden.

Las enfermedades crónicas no transmisibles son la principal causa de muerte en el mundo, con aproximadamente 41 millones de muertes anuales. Se estima que el 70 % de estas muertes ocurren en los países de bajos y medianos ingresos económicos ^1^^,^^2^. Entre las enfermedades crónicas no transmisibles, las cuatro que representan más del 80 % de todas las muertes prematuras son: las enfermedades cardiovasculares (17,9 millones), cáncer (9 millones), enfermedades respiratorias (3,9 millones) y diabetes mellitus (1,6 millones) ^3^. Estas entidades producen una alta carga de enfermedad, con una tendencia creciente con el pasar de los años. A nivel mundial, en el 2019, causaron 1,60 mil millones de años de vida ajustados en función de la discapacidad por año ^4^. Es importante considerar el alto costo económico que implican dichos años de vida perdidos y los gastos asociados a la atención médica de estas condiciones. Para el 2030, se estima un costo médico de USD$ 13 mil millones que equivale al doble del gasto generado en el 2010, calculado en USD$ 6,3 mil millones ^5^.

La transición epidemiológica actual muestra un aumento de las enfermedades crónicas no transmisibles, posiblemente como resultado de los cambios demográficos y epidemiológicos, como el envejecimiento de la población, el mayor desplazamiento urbano, los estilos de vida insalubres y la contaminación ambiental ^6^. La llegada del virus SARS-CoV2 causó un impacto multisectorial importante y puso en evidencia un efecto sindémico del virus con las enfermedades crónicas no transmisibles. Se observaron mayores tasas de infección y peores desenlaces clínicos en aquellos pacientes con antecedentes de enfermedades crónicas no transmisibles ^7^. Además, la pandemia de COVID-19 aumentó las barreras para el manejo de las enfermedades crónicas, debido a interrupciones en los servicios de salud, mayor exposición a factores de riesgo conductuales y menores probabilidades de buscar tratamiento médico por temor al contagio, lo que generó dificultades en el cumplimiento de los tratamientos y, por ende, peores desenlaces clínicos ^8^.

El tratamiento de las enfermedades crónicas no transmisibles se basa en el manejo farmacológico y no farmacológico, es decir, control de factores de riesgo y recomendaciones de estilos de vida saludables. Sin embargo, a pesar de su demostrada efectividad, la prevalencia y el control deficiente de estas enfermedades continúa en aumento ^9^. Existen múltiples elementos que contribuyen a la falta de control de las enfermedades crónicas no transmisibles, como las barreras de acceso al sistema de salud, el cumplimiento deficiente de las recomendaciones de autocuidado y el incumplimiento del tratamiento ^10^. La falta de cumplimiento terapéutico es un problema mundial que afecta principalmente a los pacientes con enfermedades crónicas no transmisibles y es, aproximadamente, del 50 % a largo plazo. Esta cifra es aún más baja en los países de medianos y bajos ingresos, lo que incurre en un menor control de la enfermedad y mayor mortalidad ^11^.

Dada la relevancia del cumplimiento del manejo farmacológico y no farmacológico, el paciente es un actor fundamental para el control de su enfermedad. Sin embargo, existen barreras personales, socioeconómicas, relacionadas con el tratamiento, la enfermedad o el sistema de salud, que impiden el cumplimiento del tratamiento y, por tanto, el control de la enfermedad ^12^.

Hasta el momento, no se dispone de revisiones sistemáticas que estimen la cifra de cumplimiento farmacológico de los pacientes colombianos con enfermedades crónicas no transmisibles. En consecuencia, el objetivo del presente estudio fue establecer la frecuencia de casos que cumplían con el tratamiento farmacológico de pacientes colombianos con enfermedades crónicas no transmisibles como hipertensión arterial, enfermedad cerebrovascular, diabetes mellitus, asma, enfermedad pulmonar obstructiva crónica y dislipidemia, entre el 2005 y el 2022.

## Materiales y métodos

### Diseño y protocolo

Se llevó a cabo una revisión sistemática con metaanálisis. Se realizó una búsqueda sistemática de la literatura, siguiendo las pautas PRISMA *(Preferred Reporting Items for Systematic Reviews and Meta-analyses)* del 2020 ^13^, para responder la pregunta: ¿cuál es la frecuencia de casos que cumplen con el tratamiento farmacológico en adultos colombianos con hipertensión arterial, enfermedad cerebrovascular, diabetes mellitus, asma, enfermedad pulmonar obstructiva crónica o dislipidemia?

### Estrategia de la búsqueda de literatura

La búsqueda se realizó en las bases de datos Medline y LILACS de forma independiente por dos investigadores. Se consideraron los reportes publicados antes del 15 febrero del 2023. Los idiomas de búsqueda fueron inglés, español y portugués. Se establecieron como términos MeSH: "Hypertension", "Diabetes mellitus, type 2", "Asthma", "Dyslipidemias", "Pulmonary disease, chronic obstructive", "Stroke", "Colombia". Se adicionaron los *entry terms* correspondientes para cada uno de los términos y se emplearon los operadores booleanos "OR" y "AND" para formar la estrategia final de búsqueda (anexo 1). Este proceso se repitió con los términos DeCS en español, inglés y portugués. Se revisaron las listas de referencias y artículos citados de literatura relevante sobre este tema para encontrar posibles estudios por incluir en esta investigación. Además, se hizo la búsqueda en Google, para evaluar la literatura gris, en donde se seleccionaron y examinaron los primeros 20 resultados.

### Criterios de elegibilidad y selección de estudios

Se incluyeron los estudios observacionales de corte transversal, las encuestas de salud y las cohortes de base poblacional, que involucraran población colombiana adulta de 18 años o más, publicados entre el 2005 y el 2022, cuyos objetivos hubieran sido estimar la frecuencia de casos que cumplían con el tratamiento farmacológico para la hipertensión arterial, la enfermedad cerebrovascular, la diabetes mellitus, el asma, la enfermedad pulmonar obstructiva crónica o la dislipidemia.

Para la definición de cumplimiento del tratamiento, se adoptó la estipulada por la Organización Mundial de la Salud (OMS): "El grado en el que la conducta de un paciente, en relación con la toma de medicación, el seguimiento de una dieta o la modificación de hábitos de vida se corresponde con las recomendaciones acordadas con el profesional sanitario" ^14^.

Los criterios de exclusión fueron: cualquier estudio no observacional (revisiones sistemáticas, ensayos clínicos, reporte de casos), estudios de casos y controles, resúmenes de congresos y cartas del editor; y participantes con condiciones específicas (mujeres en estado de gestión y niños o adolescentes con enfermedades crónicas).

### Extracción de la información

Con la plataforma Rayyan ^15^, dos autores de manera independiente excluyeron los registros duplicados y revisaron los títulos y los resúmenes de todos los estudios potencialmente relevantes para esta revisión. En la revisión de los textos completos se identificaron aquellos estudios que se debían excluir porque no estimaron el cumplimiento con el tratamiento farmacológico, tenían población no representativa o falencias en la calidad de su conducción. Las discrepancias fueron resueltas en consenso con la participación de un tercer revisor.

Se diseñó una matriz electrónica de extracción de datos mediante Google *sheets.* De manera independiente y en ciego se extrajeron las variables por parte de dos investigadores: primer autor, año de publicación, fecha de realización del estudio, diseño del estudio, tamaño muestral, ciudad o municipio, edad, media de edad, proporción de hombres y mujeres, frecuencia de casos por seco de los que cumplían con el tratamiento e instrumento utilizado para la medición del cumplimiento. Aquellos datos no proporcionados por los autores de las investigaciones fueron identificados como no disponibles y no se incluyeron en el metaanálisis. Para los estudios que evaluaron más de una enfermedad, se extrajeron los datos por separado para cada una y así se realizó el análisis. La calidad de los estudios incluidos fue evaluada de acuerdo con las directrices del *Strengthening the Reporting of Observational Studies in Epidemiology* (STROBE) ^16^. El cumplimiento de los 22 ítems puede ser consultado en el anexo 2. La calidad metodológica fue evaluada con la lista del Instituto Joanna Briggs (anexo 3 y 4) ^17^.

### Análisis estadístico

En la revisión sistemática se hizo un metaanálisis usando un modelo de efectos aleatorios para estimar la frecuencia combinada y los intervalos de confianza (IC) del 95 % mediante el método de Der Simonian y Laird ^18^. La varianza de las frecuencias fue estabilizada con la transformación del arcoseno de Freeman-Tukey ^19^. Las estimaciones se hicieron con el comando *metaprop*^20^.

La heterogeneidad estadística entre los estudios se calculó usando el estadístico I2 (índice de inconsistencia). Al ser un metaanálisis de proporciones, el nivel de heterogeneidad es usualmente alto por la naturaleza de los datos, el tiempo, el lugar y las diferentes enfermedades evaluadas. Por ende, no se definieron puntos de corte como se hace en los metaanálisis de intervención ^21^.

El sesgo de publicación fue evaluado visualmente con el gráfico de embudo y la estimación del efecto de estudios de tamaño de muestra pequeño se evaluó con la prueba de Egger. El análisis fue procesado en el paquete estadístico Stata™, versión 15.1, y en Microsoft Office Excel 2013. El nivel de significancia fue del 5 %.

## Resultados

### Proceso de selección de los artículos

**Figura 1:**
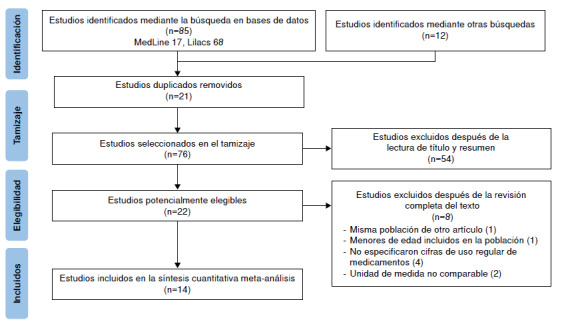
Proceso de selección de artículos

La búsqueda identificó 85 publicaciones en las bases de datos definidas. Estas se unificaron en el aplicativo web RAYYAN QCRI, y se eliminaron 20 duplicados. Los 65 estudios restantes se evaluaron por título y resumen. Se excluyeron 43 estudios que no se ajustaban a la pregunta de interés, se seleccionaron 22 artículos para determinar su elegibilidad final y de estos, se excluyeron ocho estudios tras la revisión completa del texto. Finalmente, se incluyeron 14 artículos en esta revisión sistemática que cumplían los criterios de inclusión. El diagrama de PRISMA completo se presenta en la figura 1.

### Características de los estudios incluidos y sus participantes

Los 14 artículos seleccionados fueron caracterizados por año de realización del estudio, revista de publicación, ubicación del estudio, tipo de estudio, enfermedad, población total, población que cumplía con el tratamiento farmacológico y el método de medición del cumplimiento. El cuadro 1 resume las principales características de los estudios. Se incluyó un total de 5.658 pacientes con una edad media entre los 33,7 y los 70 años.

**Cuadro 1:** Características sociodemográficas de la población de los estudios seleccionados

Autor	Año	Revista	Ubicación	Tipo de estudio	Enfermedad	Población			Edad media	Casos que cumplían			Medición del cumplimiento
						total	H	M		Total	H	M	
Ospina *et al*. ^22^	2016	Arch Med	Municipal: Bucaramanga	Transversal	Diabetes mellitus	411	96	315	62,25	376	ND	ND	CMG
Falun *et al*. ^23^	2017-2019	Acta Méd. Colomb	Municipal: Dosquebradas	Transversal	Diabetes mellitus	83	24	59	33,7	51	ND	ND	CMG
Rodríguez *et al*. ^24^	2010	Rev Fac Nac Salud Pública	Municipal: Cali	Transversal	Diabetes mellitus- Hipertensión arterial	Hipertensión arterial: 202 Diabetes mellitus: 32 Hipertensión arterial- Diabetes mellitus: 43	97	180	56,5	258	ND	ND	CAT-hipertensión arterial
Martínez- Domínguez *et al*. ^25^	2011	Arch Med	Municipal: Medellín	Transversal	Diabetes mellitus	70	24	46	61,9	56 ND		ND	SDSCA
Machado *et al*. ^26^	2010-2011	Rev Panam Salud Pública	Nacional: Bogotá, Barranquilla, Bucaramanga, Cali, Cartagena, Ibagué, Manizales, Medellín, Pereira, y Santa Marta	Transversal	Dislipidemia	600	24	263	65,91	420	ND	ND	Cumplimiento de recomendaciones
Arciniegas *et al*. ^27^	2009	Rev Univ Ind Santander Salud	Municipal: Pereira	Cohorte	Enfermedad pulmonar obstructiva crónica	215	101	114	70	61	ND	ND	Número de cilindros utilizados al mes
Quiroz *et al.*^28^	2015	Rev Chil Salud Pública	Nacional: Bogotá, Medellín, Quibdó	Transversal	Hipertensión arterial	258	97	161	58	132	ND	ND	CMG y CMBG
Guarin *et al.*^29^	2013- 2014	Rev Fac Med (Bogotá)	Municipal: Bogotá	Transversal	Hipertensión arterial	242	82	160	ND	76	ND	ND	CMG
Benavides *et al.*^30^	2011	Univ Salud	Municipal: Pasto	Transversal	Hipertensión arterial	128	40	88	ND	53	14	39	CMG
Casas *et al.*^31^	2010- 2011	Hacia Promoc Salud	Departamental: Caldas	Transversal	Hipertensión arterial	295	71	224	64,7	133	ND	ND	CMG
Castaño *et al.*^32^	2011	Rev Fac Med	Municipal: Manizales	Transversal	Hipertensión arterial	200	53	147	63,76	90	ND	ND	CMG y CMBG
Martínez *et al.*^33^	2009	Rev Fac Nac Salud Pública	Departamental: Risaralda	Transversal	Hipertensión arterial	133	27	106	62	56	9	47	CMBG
Herrera *et al*. ^34^	2004	Rev Colomb Cardiol	Departamental: Cali y municipios aledaños	Transversal	Hipertensión arterial	356	128	228	61,2	200	ND	ND	CMG
Camacho *et al.*^35^	2005- 2009	Gobal Heart	Nacional: Atlántico, Bolívar, Cesar, Caldas,	Cohorte	Hipertensión arterial	1.661	454	1200	50,7	1.511	ND	ND	Cumplimiento del tratamiento autoinformado
					Diabetes mellitus	422	137	285		324	ND	ND	
					Evento cerebrovascular	109	30	79		35	ND	ND	
					Asma	198	49	149		63	ND	ND	

H: hombres; M: mujeres; ND: datos no disponibles; CMG: cuestionario Morisky-Green; CAT-hipertensión arterial: cuestionario de cumplimiento del tratamiento para la hipertensión arterial; SDSCA: cuestionario resumen de actividades de autocuidado de la diabetes; CMBG: cuestionario Martin Bayarre-Grau

La mayoría de los artículos fueron publicados en revistas científicas nacionales, excepto tres que se publicaron en revistas internacionales. Con respecto a la ubicación del estudio, ocho fueron a nivel municipal ^22^^-^^25^^,^^27^^,^^29^^,^^30^^,^^32^, tres a nivel departamental ^31^^,^^33^^,^^34^ y tres a nivel nacional ^26^^,^^28^^,^^35^. De los 14 estudios, 12 tenían un diseño transversal y 2 eran de cohortes ^27^^,^^35^.

La enfermedad crónica más estudiada fue la hipertensión arterial (nueve estudios, 61 % pacientes), seguida de diabetes mellitus (dos estudios, 18 % pacientes), y dislipidemia (11 % pacientes), enfermedad pulmonar obstructiva crónica (3,8 % pacientes), asma (3,5 % pacientes), enfermedad cerebrovascular (109 pacientes) y diabetes mellitus con hipertensión (43 pacientes) con un estudio cada una.

El tamaño de muestra más grande fue de 2.390 pacientes ^35^ y el más pequeño de 70 pacientes ^25^. En todos los estudios se encontró que había preponderancia de las mujeres frente a los hombres (71,3 % de mujeres y 28,7 % de hombres). Con respecto al método de medición del cumplimiento del tratamiento todos fueron de autorreporte, y los cuestionarios de Morisky-Green y Martin-Bayarre-Grau los más utilizados (anexo 5).

### Cumplimiento del tratamiento farmacológico

**Figura 2:**
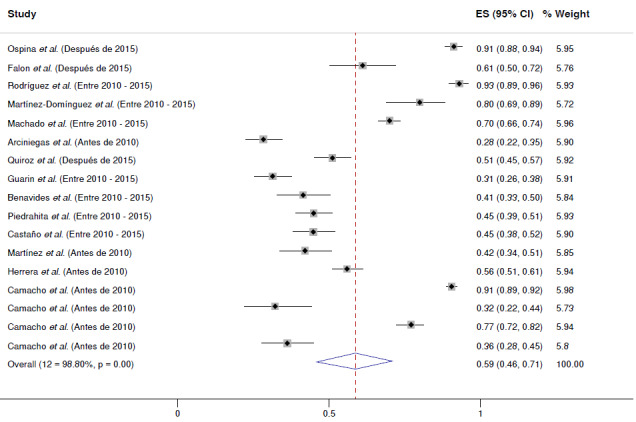
Frecuencia de caso que cumplían con el tratamiento farmacológico de enfermedades crónicas en población colombiana entre el 2005-2022. La prevalencia global del uso de medicamentos en Colombia es del 59 % (IC_95%_ = 46-71 %) con un 12 del 98,8 % (p<0,001).

En general, el cumplimiento del tratamiento farmacológico fue del 59 %, con una heterogeneidad I2 del 98,8 % (IC_95%_ = 46-71 %). El menor porcentaje de cumplimiento del tratamiento encontrado en los estudios fue del 28,37 % para la enfermedad pulmonar obstructiva crónica por Arciniegas *et al.*^27^, y la cifra más alta de cumplimiento del tratamiento fue del 93 % para la diabetes mellitus con hipertensión arterial en el estudio de Rodríguez *et al.*^24^, seguido de un cumplimiento del tratamiento del 91 % para la hipertensión arterial en el de Camacho *et al.*^35^ y para la diabetes mellitus en el de Ospina *et al.*^22^ (figura 2). El gráfico de embudo es simétrico; sin embargo, la prueba de Egger sugiere un riesgo de sesgo de publicación relacionado con los estudios seleccionados (p=0,004) (anexo 6 y 7).

### Análisis de subgrupos

Por tipo de enfermedad crónica, se encontró que el artículo de Rodríguez *et al.*^24^ no diferenciaba cumplimiento del tratamiento farmacológico en los pacientes con diabetes mellitus o hipertensión arterial, solo reportaron un cumplimiento general del 93 % (IC_95%_ = 89-96 %), la mayor descrita en la revisión. Al no tener más datos en este subgrupo, no se hizo el metaanálisis, solo se reportaron sus valores, que influyeron en la ponderación final. La segunda cifra más alta fue para la diabetes mellitus, con una frecuencia general de casos que cumplían con el tratamiento farmacológico del 79 % (IC_95%_ = 65-90 %; 12=94,5 %, p<0,001). Solo uno de los artículos incluyó pacientes con dislipidemia, con un cumplimiento del tratamiento del 70 % (IC_95%_ = 66-74 %) ^26^, la enfermedad pulmonar obstructiva crónica con un cumplimiento del tratamiento con oxígeno del 28 % (IC_95%_ = 22-35 %) ^26^, la enfermedad cardiovascular con un cumplimiento del tratamiento del 32 % (IC_95%_ = 22-44 %) ^35^ y el asma con un cumplimiento del tratamiento del 36 % (IC_95%_ = 28-45 %) ^35^. Al igual que en el subgrupo de diabetes mellitus e hipertensión arterial, en estas cuatro enfermedades no se hizo un submetaanálisis, aunque sus pesos fueron usados para la ponderación final.

**Figura 3:**
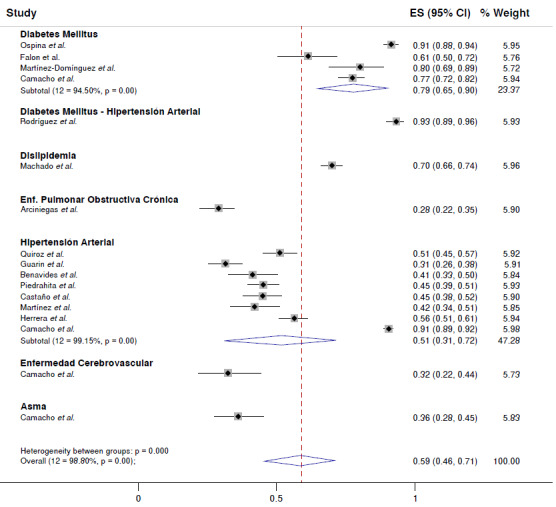
Cumplimiento del tratamiento farmacológico, según subgrupo de enfermedad crónica

Por otro lado, la hipertensión arterial fue la entidad con mayor cantidad de artículos, con una media de cumplimiento del tratamiento del 51 % (IC_95y_ = 31-72%; 12=99,15%, p<0,001) (figura 3).

**Figura 4:**
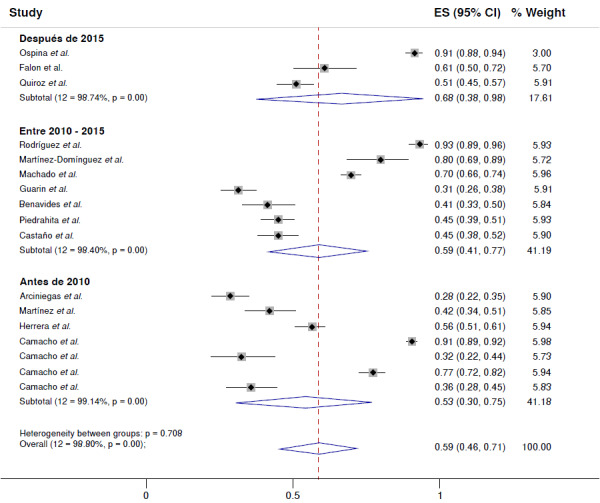
Cumplimiento del tratamiento farmacológico, según subgrupo de año de realización del estudio

Por fecha de realización del estudio, se observó que el cumplimiento del tratamiento ha aumentado con el tiempo, pasando del 53 % (IC_95%_ = 3075 %; 12=99,14 %, p<0,001) en los artículos previos al 2010, al 59 % (IC_95%_ = 41-77 %; I2 =98.40 %, p<0,001) en los artículos entre el 2010 y el 2015, hasta el 70 % (IC_95%_ = 3795 %; 12=98,74 %, p<0,001) después del 2015 (figura 4).

## Discusión

Este artículo presenta los resultados de una revisión sistemática y un metaanálisis de 14 estudios que incluyeron 5.658 pacientes, en el que se encontró que el cumplimiento del tratamiento para enfermedades crónicas no transmisibles en Colombia es del 59 %. Este es el primer estudio de este tipo que se realiza en población colombiana.

Lemstra *et al.* publicaron en el 2018 un metaanálisis sobre el incumplimiento farmacológico en enfermedades crónicas, que reportó una tasa de incumplimiento para los antihipertensivos, los hipolipemiantes, los antidiabéticos y los antidepresivos del 14,6 % (IC_95%_ = 13,1-16,2 %). Según Lemstra, el bajo cumplimiento del tratamiento fue mayor para los medicamentos hipolipemiantes (20,8 %; IC_95%_ = 16,0-25,6 %), seguido de los antihipertensivos (12,4 %; IC_95%_ = 9,5-15,3 %) y los antidiabéticos (13,2 %; IC_95%_ = 9,616,8 %) (35). Los resultados del presente estudio son similares a estos, pero en contraste con Lemstra *et al.,* la dislipidemia fue la segunda enfermedad con mayor cumplimiento del tratamiento, precedida por la diabetes mellitus ^36^.

De las enfermedades crónicas no transmisibles incluidas en el estudio, la diabetes mellitus fue la entidad con la cifra más alta de cumplimiento del tratamiento, superior a la encontrada en el metaanálisis de Piragine *et al.,* que reporta cifras de cumplimiento del tratamiento a los antidiabéticos orales del 54 % (IC 95 %: 51-58 %) ^37^. A diferencia del presente estudio, Piragine *et al.* incluyeron reportes que analizaron pacientes con diabetes mellitus y otras comorbilidades, por lo que se aumentó el número de pacientes con polifarmacia y con esto, disminuyó el cumplimiento del tratamiento ^38^. De los estudios incluidos en este análisis, el de Ospina *et al.*^22^ fue el que reportó mayor cumplimiento del tratamiento (91 %) de los pacientes diabéticos, pero únicamente el 3,65 % tenía un cumplimiento alto y el resto, un cumplimiento medio. Otro metaanálisis realizado por Azharuddin *et al,* en países de bajos y medianos ingresos, encontró que el 43,3 % (IC _95%_ = 17.569.4 %, p<0,001) y el 29,1 % (IC_95%_ = 19,8-38,4 p<0,001) de los pacientes no cumplían con el tratamiento igual o mayor del 80-90 % ^39^.

La dislipidemia fue la segunda enfermedad con mayor cumplimiento del tratamiento (70 %), pero estos resultados son de un solo estudio que, a pesar de incluir pacientes de diez departamentos de Colombia, no se pueden extrapolar totalmente. Lo anterior muestra la falta de investigación en esta enfermedad. Según Machado *et al.*^26^, el 69,1 % de la población logró la meta de reducir el colesterol de baja densidad y el 70,5 % redujo el colesterol total, lo que sustenta los resultados del cumplimiento del tratamiento. No obstante, en los grupos de bajo riesgo estas cifras fueron mucho menores de lo esperado, a pesar de lo que es preocupante porque la población del estudio cuenta con fácil acceso a los medicamentos.

En otro metaanálisis realizado por Lemstra *et al.* se evidenció una cifra de cumplimiento del tratamiento similar a la encontrada, con un cumplimiento del 49 % (IC_95%_ = 48,9-49,2 %) en el uso de estatinas al año de seguimiento en los estudios observacionales, y del 90,3 % (IC_95%_ = 89,8-90,8 %) en los ensayos clínicos aleatorizados. Además, encontraron que los pacientes que utilizaban estatinas como prevención primaria tienen menor cumplimiento del tratamiento, posiblemente porque los individuos pueden sentir que no existe una amenaza inmediata para su salud ^40^.

La evidencia relacionada con la hipertensión arterial fue la más contundente del presente análisis, con nueve estudios incluidos y 3.475 pacientes analizados. Abegaz *et al.,* en el 2017, encontraron que el 45,2 % (IC_95%_ = 34,4-56,1 %, p<0,001) de los pacientes hipertensos y el 31,2 % de los hipertensos con comorbilidades no cumplían con el tratamiento ^41^. Esto es comparable con las cifras encontradas en el metaanálisis realizado, en el que el 51 % de los hipertensos cumplieron con el tratamiento farmacológico.

Resultados similares se han encontrado en las revisiones de Nielsen *et al.* -que reportan un incumplimiento del tratamiento farmacológico en hipertensos del 63,35 % (IC _95%_ = 38,78-87,91 %) ^42^- y Lemstra *et al.* que al año del tratamiento antihipertensivo encontró un cumplimiento del 48,5 % (IC_95%_ = 47,7-49,2 %) ^43^. Del presente estudio, el artículo de Rodríguez *et al.* fue el que mostró mayor cumplimiento de los pacientes con hipertensión arterial o diabetes mellitus (93 %), una cifra mayor a la reportada en la literatura. No obstante, este artículo menciona que los participantes presentaron cifras tensionales sistólicas (35 %) y diastólicas (20 %) fuera de metas ^24^. Esto demuestra la necesidad e importancia de la observancia de las medidas no farmacológicas en el control de la enfermedad. Cabe recalcar que los pacientes incluidos en este estudio hacían parte de un programa de protección renal, lo que podría incidir en una mayor conciencia de la enfermedad. Además, existe un alto riesgo de sesgo de la información dado que no se utilizó un cuestionario validado sino el autorreporte ^24^.

Para evaluar el cumplimiento del tratamiento de los pacientes con asma (36 %), enfermedad cardiovascular (32 %) y enfermedad pulmonar obstructiva crónica (28 %), únicamente se incluyó un estudio por patología y fueron las que tuvieron menor cumplimiento del tratamiento farmacológico ^35^. Con respecto al asma, la mayoría de la literatura se centra en la población menor de 18 años y la efectividad de las intervenciones. En la revisión sistemática de Marjolein *et al.* se observó que el cumplimiento del tratamiento en general fue baja en la población pediátrica y adulta. En los niños, la media del cumplimiento del tratamiento fue solo del 20 al 33,9 % para los corticosteroides inhalados, y en adultos, varió entre el 15 y el 54 % ^44^. En este caso se deben tener en cuenta las dificultades adicionales propias de la terapia inhalada que pueden favorecer el incumplimiento del tratamiento ^45^. Para la enfermedad pulmonar obstructiva crónica y la enfermedad cardiovascular, se encontraron mayores cifras de cumplimiento del tratamiento: 46,3 % de los pacientes tenía un nivel moderado de la terapia inhalada para la enfermedad pulmonar obstructiva crónica, el 41,6 % de los pacientes tenía un nivel alto de la terapia oral y 64,1 % de los participantes con ictus tuvieron un cumplimiento alto de la medicación (IC_95%_ = 57,470,8 %) ^45^^,^^46^.

En todos los estudios incluidos, la proporción de mujeres fue mayor que la de los hombres, pero no se logró hacer la estimación de cumplimiento del tratamiento por sexo debido a que solo dos artículos hicieron el análisis para hombres y mujeres hipertensos. Benavidez *et al.*^30^ reportaron un mayor cumplimiento del tratamiento con los medicamentos antihipertensivos en las mujeres (44 %) respecto a los hombres (35 %), similar a lo encontrado por Martinez *et al.* (44 % mujeres, 33 % hombres) ^33^. Esto contradice los resultados del metaanálisis de Abegaz *et al.* que reportó un porcentaje de incumplimiento mayor en mujeres hipertensas (53,9 %, IC_95%_ = 49,757,9 %, p<0,001) que en hombres hipertensos (46,2 %, IC_95_% = 42,2-50,2 %, p=0,020) ^41^.

Además de las escasas herramientas disponibles para evaluar el cumplimiento del tratamiento, pocas han sido objetivamente validadas en la población colombiana. Entre ellas está la escala de cumplimiento de la medicación de Morisky-8 (MMAS-8), la herramienta de autorreporte más reconocida en el ámbito de la investigación del cumplimiento del tratamiento, usada principalmente por pacientes hipertensos y la más utilizada en los estudios incluidos en este metanálisis ^41^. Esta escala ha sido validada nacionalmente, en pacientes hipertensos y con enfermedad renal crónica ^47^^-^^49^. Algunos autores han indagado la validez de las medidas de autoinforme para evaluar el cumplimiento del tratamiento farmacológico, pues a pesar de su bajo costo, rapidez y facilidad de aplicación, tienen mayor propensión de manipulación por parte del paciente, dando lugar a una baja sensibilidad comparada con las medidas objetivas (evolución clínica, niveles de medicamentos en sangre u orina, niveles de presión arterial, etc.) y los sistemas electrónicos de monitoreo *(Medication Event Management Systems,* MEMS) que son el patrón de referencia ^50^^-^^52^. Por esto, es fundamental disponer de instrumentos de medición confiables que combinen la asequibilidad del autoinforme con la mayor sensibilidad del monitoreo electrónico para mejorar la validez de los datos.

La falta de cumplimiento del tratamiento de los pacientes con enfermedades crónicas es un tema de preocupación dada su asociación con resultados adversos de la enfermedad cardiovascular, pues es la consecuencia principal de un control inadecuado de la presión arterial ^53^. En un metaanálisis, Arbeláez *et al.* encontraron que la falta de cumplimiento al tratamiento farmacológico era mayor (83,7 %) en aquellos pacientes con presión arterial no controlada, comparada con los hipertensos controlados (59,7 %) ^41^. En los pacientes hipertensos, el reducir la presión arterial sistólica en 10 mm Hg, disminuía un 20 % el riesgo de eventos cardiovasculares graves; un 17 % enfermedad coronaria; un 27 % enfermedad cardiovascular; un 28 % insuficiencia cardiaca y un 13 % la mortalidad por cualquier causa ^54^. En los pacientes diabéticos, la incumplimiento con la medicación se relacionó con mayores niveles de hemoglobina glicosilada, presión arterial sistólica, diastólica y colesterol de baja densidad, mayor hospitalización por todas las causas (OR=1,58; p<0,001) y mayor mortalidad por todas las causas (OR=1,81; p<0,001) ^55^.

El cumplimiento del tratamiento farmacológico de las enfermedades crónicas no transmisibles depende de múltiples factores: creencias, nivel socioeconómico y educativo, conocimiento de la enfermedad, raza y relación médico-paciente, entre otros factores ^39^^,^^56^^,^^57^. Se ha visto que una de las barreras más importantes para el cumplimiento son los sistemas de salud para acceder a la medicación y, en consecuencia, el gasto económico que esto acarrea ^58^. Por lo tanto, la identificación de estos obstáculos permitiría mejorar el cumplimiento del tratamiento, reducir los costos, optimizar el tratamiento y lograr un control adecuado de la enfermedad. Debido a la complejidad y a los diversos factores que afectan el cumplimiento del tratamiento, las intervenciones más efectivas deben ser multicomponente y estar adaptadas a cada individuo, como las educativas, el desarrollo de planes de tratamiento individualizados, la intervención sobre las barreras, el apoyo continuo, las entrevistas motivacionales y el seguimiento estricto ^50^^,^^59^^,^^60^.

Se debe tener en cuenta que los resultados de este metaanálisis proporcionan una estimación general de acuerdo con los estudios incluidos, pero puede variar según la enfermedad, la región o los grupos poblacionales dentro de Colombia. Lo anterior, se correlaciona con la alta heterogeneidad encontrada -esperada en los metaanálisis de proporción- posiblemente por las diferencias en las características sociodemográficas de las poblaciones, las metodologías de los estudios, el tamaño muestral y las diferentes enfermedades evaluadas. Por otro lado, se debe tener en cuenta el sesgo de información de cada estudio, pues en su mayoría se utilizaron cuestionarios de autorreporte. Además, la mayor parte de la literatura se relaciona con la hipertensión arterial mientras que hay poca evidencia sobre la enfermedad pulmonar obstructiva crónica, el asma, la enfermedad cardiovascular y la dislipidemia. Por último, aunque el gráfico de embudo es simétrico, la prueba de Egger muestra un valor que podría sugerir sesgo de publicación, pero se debe tener en cuenta que la alta heterogeneidad del tipo de estudios incluidos puede influenciar esta medida estadística. Por lo tanto, su interpretación debe ser cautelosa y actualmente no hay un consenso para evaluar la heterogeneidad de los metaanálisis de frecuencias.

En conclusión, esta revisión sistemática muestra un bajo cumplimiento del tratamiento farmacológico de las enfermedades crónicas no transmisibles, lo que puede repercutir en los resultados clínicos y en la carga de enfermedad a largo plazo. Es necesario llevar a cabo una mayor cantidad de investigaciones en el contexto local, con el objetivo de plantear intervenciones poblacionales y con esto lograr un adecuado control de las enfermedades crónicas no transmisibles.

## Archivos suplementarios

Anexo 1. Estrategia de búsqueda en Medline y LILACS

**Medline:** ((((((((((((((((((((((((((((((((((Diabetes Mellitus, Type 2[MeSH Terms]) OR (Diabetes Mellitus, Type 2[Text Word])) OR (Diabetes Mellitus, Noninsulin-Dependent[Text Word])) OR (Diabetes Mellitus, Ketosis-Resistant[Text Word])) OR (Diabetes Mellitus, Ketosis Resistant[Text Word])) OR (Ketosis-Resistant Diabetes Mellitus[Text Word])) OR (Diabetes Mellitus, Non Insulin Dependent[Text Word])) OR (Diabetes Mellitus, Non-Insulin-Dependent[Text Word])) OR (Non-Insulin-Dependent Diabetes Mellitus[Text Word])) OR (Diabetes Mellitus, Stable[Text Word])) OR (Stable Diabetes Mellitus[Text Word])) OR (Diabetes Mellitus, Type II[Text Word])) OR (NIDdiabetes mellitus[Text Word])) OR (Diabetes Mellitus, Noninsulin Dependent[Text Word])) OR (Diabetes Mellitus, Maturity-Onset[Text Word])) OR (Diabetes Mellitus, Maturity Onset[Text Word])) OR (Maturity-Onset Diabetes Mellitus[Text Word])) OR (Maturity Onset Diabetes Mellitus[Text Word])) OR (MODY[Text Word])) OR (Diabetes Mellitus, Slow-Onset[Text Word])) OR (Diabetes Mellitus, Slow Onset[Text Word])) OR (Slow-Onset Diabetes Mellitus[Text Word])) OR (Type 2 Diabetes Mellitus[Text Word])) OR (Noninsulin-Dependent Diabetes Mellitus[Text Word])) OR (Noninsulin Dependent Diabetes Mellitus[Text Word])) OR (Maturity-Onset Diabetes[Text Word])) OR (Diabetes, Maturity-Onset[Text Word])) OR (Maturity Onset Diabetes[Text Word])) OR (Type 2 Diabetes[Text Word])) OR (Diabetes, Type 2[Text Word])) OR (Diabetes Mellitus, Adult-Onset[Text Word])) OR (Adult-Onset Diabetes Mellitus[Text Word])) OR (Diabetes Mellitus, Adult Onset[Text Word])) OR ((((((Asthma[MeSH Terms]) OR (Asthma[Text Word])) OR (Asthmas[Text Word])) OR (Bronchial Asthma[Text Word])) OR (Asthma, Bronchial[Text Word])) OR (((((((Dyslipidemias[MeSH Terms]) OR (dyslipidemia[MeSH Terms])) OR (Dyslipoproteinemias[MeSH Terms])) OR (Dyslipoproteinemia[MeSH Terms])) OR (Dyslipidemias[Text Word])) OR (Dyslipidemia[Text Word])) OR (Dyslipoproteinemias[Text Word])) OR (Dyslipoproteinemia[Text Word]) OR (((((((((((((Pulmonary Disease, Chronic Obstructive[MeSH Terms]) OR (Pulmonary Disease, Chronic Obstructive[Text Word])) OR (Chronic Obstructive Lung Disease[Text Word])) OR (Chronic Obstructive Pulmonary Diseases[Text Word])) OR (COAD[Text Word])) OR (COPD[Text Word])) OR (Chronic Obstructive Airway Disease[Text Word])) OR (Chronic Obstructive Pulmonary Disease[Text Word])) OR (Airflow Obstruction, Chronic[Text Word])) OR (Airflow Obstructions, Chronic[Text Word])) OR (Chronic Airflow Obstructions[Text Word])) OR (Chronic Airflow Obstruction[Text Word])) OR (((((((((((((((((((((((((((((((stroke[MeSH Terms]) OR (stroke[Text Word])) OR (Strokes[Text Word])) OR (Cerebrovascular Accident[Text Word])) OR (Cerebrovascular Accidents[Text Word])) OR (CVA (Cerebrovascular Accident[Text Word]))) OR (CVAs (Cerebrovascular Accident[Text Word]))) OR (Cerebrovascular Apoplexy[Text Word])) OR (Apoplexy, Cerebrovascular[Text Word])) OR (Vascular Accident, Brain[Text Word])) OR (Brain Vascular Accident[Text Word])) OR (Brain Vascular Accidents[Text Word])) OR (Vascular Accidents, Brain[Text Word])) OR (Cerebrovascular Stroke[Text Word])) OR (Cerebrovascular Strokes[Text Word])) OR (Stroke, Cerebrovascular[Text Word])) OR (Strokes, Cerebrovascular[Text Word])) OR (Apoplexy[Text Word])) OR (Cerebral Stroke[Text Word])) OR (Cerebral Strokes[Text Word])) OR (Stroke, Cerebral[Text Word])) OR (Strokes, Cerebral[Text Word])) OR (Stroke, Acute[Text Word])) OR (Acute Stroke[Text Word])) OR (Acute Strokes[Text Word])) OR (Strokes, Acute[Text Word])) OR (Cerebrovascular Accident, Acute[Text Word])) OR (Acute Cerebrovascular Accident[Text Word])) OR (Acute Cerebrovascular Accidents[Text Word])) OR (Cerebrovascular Accidents, Acute[Text Word])) AND (((((((((((((((((((((((((((((((((((((((((((((((((((((((Medication Adherence[MeSH Terms]))) OR (Medication Adherence[Text Word]) ) OR (Adherence, Medication[Text Word])) OR (Drug Adherence[Text Word])) OR (Adherence, Drug[Text Word])) OR (Medication Nonadherence[Text Word])) OR (Nonadherence, Medication[Text Word])) OR (Medication Noncompliance[Text Word])) OR (Noncompliance, Medication[Text Word])) OR (Medication Non-Adherence[Text Word])) OR (Medication Non Adherence[Text Word])) OR (Non-Adherence, Medication[Text Word])) OR (Medication Persistence[Text Word])) OR (Persistence, Medication[Text Word])) OR (Medication Compliance[Text Word])) OR (Compliance, Medication[Text Word])) OR (Medication Non-Compliance[Text Word])) OR (Medication Non Compliance[Text Word])) OR (Non-Compliance, Medication[Text Word])) OR (Drug Compliance[Text Word])) OR (Compliance, Drug[Text Word])) OR (Patient Compliance[MeSH Terms])) OR (Patient Compliance[Text Word])) OR (Compliance, Patient[Text Word])) OR (Patient Adherence[Text Word])) OR (Adherence, Patient[Text Word])) OR (Patient Cooperation[Text Word])) OR (Cooperation, Patient[Text Word])) OR (Client Compliance[Text Word])) OR (Client Compliances[Text Word])) OR (Compliance, Client[Text Word])) OR (Client Adherence[Text Word])) OR (Adherence, Client[Text Word])) OR (Treatment Compliance[Text Word])) OR (Compliance, Treatment[Text Word])) OR (Treatment Compliances[Text Word])) OR (Therapeutic Compliance[Text Word])) OR (Compliance, Therapeutic[Text Word])) OR (Therapeutic Compliances[Text Word])) OR (Patient Non-Compliance[Text Word])) OR (Non-Compliance, Patient[Text Word])) OR (Patient Non Compliance[Text Word])) OR (Patient Noncompliance[Text Word])) OR (Noncompliance, Patient[Text Word])) OR (Patient Nonadherence[Text Word])) OR (Nonadherence, Patient[Text Word])) OR (Non-Adherent Patient[Text Word])) OR (Non Adherent Patient[Text Word])) OR (Non-Adherent Patients[Text Word])) OR (Patient, Non-Adherent[Text Word])) OR (Patient Non-Adherence[Text Word])) OR (Non-Adherence, Patient[Text Word])) OR (Patient Non Adherence[Text Word]))) AND ((colombia[MeSH Terms]) OR (colombia[Text Word])) OR ((((((((((((((hypertension[MeSH Terms]) OR (essential hypertension[MeSH Terms])) OR (hypertension[Text Word])) OR (essential hypertension[Text Word])) OR (Blood Pressure, High[Text Word])) OR (Blood Pressures, High[Text Word])) OR (High Blood Pressure[Text Word])) OR (High Blood Pressures[Text Word])) OR (Primary Hypertension[Text Word])) OR (Hypertension, Primary[Text Word])) OR (Hypertensions, Primary[Text Word])) OR (Primary Hypertensions[Text Word])) OR (Hypertension, Essential[Text Word]))

**LILACS:** ((Diabetes Mellitus Tipo 2) OR (diabetes mellitusIM) OR (diabetes mellitusNID) OR (Diabetes Mellitus Estable) OR (Diabetes) OR (Mellitus Resistente a la Cetosis) OR (Diabetes Mellitus de Inicio Adulto) OR (Diabetes Mellitus de Inicio Lento) OR (Diabetes Mellitus de Inicio en la Madurez) OR (Diabetes Mellitus no Insulino-Dependiente) OR (Diabetes Mellitus no Insulinodependiente) OR (Diabetes Tipo 2) OR (Diabetes Mellitus, Type 2 ) OR (Adult-Onset Diabetes Mellitus) OR (Diabetes Mellitus, Adult Onset) OR (Diabetes Mellitus, Adult-Onset) OR (Diabetes Mellitus, Ketosis Resistant) OR (Diabetes Mellitus, Ketosis-Resistant) OR (Diabetes Mellitus, Maturity Onset) OR (Diabetes Mellitus, Maturity-Onset) OR (Diabetes Mellitus, Non Insulin Dependent) OR (Diabetes Mellitus, Non-Insulin-Dependent) OR (Diabetes Mellitus, Noninsulin Dependent) OR (Diabetes Mellitus, Noninsulin-Dependent) OR (Diabetes Mellitus, Slow Onset) OR (Diabetes Mellitus, Slow-Onset) OR (Diabetes Mellitus, Stable) OR (Diabetes Mellitus, Type II) OR (Diabetes, Maturity-Onset) OR (Diabetes, Type 2) OR (Ketosis-Resistant Diabetes Mellitus) OR (MODY) OR (Maturity Onset Diabetes) OR (Maturity Onset Diabetes Mellitus) OR (Maturity-Onset Diabetes) OR (Maturity-Onset Diabetes Mellitus) OR (NIDdiabetes mellitus) OR (Non-Insulin-Dependent Diabetes Mellitus) OR (Noninsulin Dependent Diabetes Mellitus) OR (Noninsulin-Dependent Diabetes Mellitus) OR (Slow-Onset Diabetes Mellitus) OR (Stable Diabetes Mellitus) OR (Type 2 Diabetes) OR (Type 2 Diabetes Mellitus) OR (Diabetes Mellitus Estável) OR (Diabetes Mellitus Resistente a Cetose) OR (Diabetes Mellitus de Início Gradativo) OR (Diabetes Mellitus de Início na Maturidade) OR (Diabetes Mellitus de Início no Adulto) OR (Diabetes Mellitus não Dependente de Insulina) OR (Diabetes Mellitus não Insulino-Dependente) OR (Diabetes Mellitus não Insulinodependente) OR (Diabetes do Tipo 2)) OR ((Asma) OR (Asma Bronquial) OR (Asthma) OR (Asthma, Bronchial) OR (Asthmas) OR (Bronchial Asthma) OR (Asma Brônquica)) OR ((Dislipidemias) OR (Dyslipidemias) OR (Dislipidemias) OR (Dislipidemias) OR (Dislipoproteinemias)) OR ((Enfermedad Pulmonar Obstructiva Crónica) OR (COAD) OR (enfermedad pulmonar obstructiva crónica) OR (EVOC) OR (Enfermedad Obstructiva Crónica de las Vías Aéreas) OR (Enfermedad Pulmonar Crónica Obstructiva) OR (Enfermedad del Pulmón Crónica Obstructiva) OR (Neumopatía Obstructiva Crónica) OR (Obstrucción Crónica del Flujo Aéreo) OR (Obstrucción del Flujo Aéreo Crónica) OR (Pulmonary Disease, Chronic Obstructive) OR (Airflow Obstruction, Chronic) OR (Airflow Obstructions, Chronic) OR (COPD) OR (Chronic Airflow Obstruction) OR (Chronic Airflow Obstructions) OR (Chronic Obstructive Airway Disease) OR (Chronic Obstructive Lung Disease) OR (Chronic Obstructive Pulmonary Disease) OR (Doença Pulmonar Obstrutiva Crônica) OR (DPOC) OR (Doença Obstrutiva Crônica Pulmonar) OR (Doença Obstrutiva Crônica das Vias Aéreas) OR (Doença Obstrutiva Crônica do Pulmão) OR (Obstrução Crônica do Fluxo Respiratório) OR (Obstrução do Fluxo Respiratório Crônica)) OR ((hipertension) or (Presión Sanguínea Alta) or (Hypertension) or (Blood Pressure, High) or (Blood Pressures, High) or (High Blood Pressure) or (High Blood Pressures) or (Hipertensão) or (Hipertensão Arterial) or (Hipertensão Arterial Sistêmica) or (Pressão Arterial Alta) or (Pressão Sanguínea Alta) or (Hypertension artérielle) or (Hypertension) or (Hypertension chronique) or (Hypertension permanente)) OR ((Accidente Cerebrovascular) OR (Accidente Cerebral Vascular) OR (Accidente Cerebrovascular Agudo) OR (Accidente Vascular Cerebral) OR (Accidente Vascular del Cerebro) OR (Accidente Vascular Encefálico) OR (Accidentes Cerebrovasculares) OR (ACV Agudo) OR (Apoplejía) OR (Apoplejía Cerebral) OR (Apoplejía Cerebrovascular) OR (Ataque) OR (Ataque Cerebral) OR (Ataque Cerebrovascular) OR (Ataque Cerebrovascular Agudo) OR (AVC) OR (AVE) OR (Ictus) OR (Ictus Cerebral) OR (Stroke) OR (Acute Cerebrovascular Accident) OR (Acute Cerebrovascular Accidents) OR (Acute Stroke) OR (Acute Strokes) OR (Apoplexy) OR (Apoplexy, Cerebrovascular) OR (Brain Vascular Accident) OR (Brain Vascular Accidents) OR (Cerebral Stroke) OR (Cerebral Strokes) OR (Cerebrovascular Accident) OR (Cerebrovascular Accident, Acute) OR (Cerebrovascular Accidents) OR (Cerebrovascular Accidents, Acute) OR (Cerebrovascular Apoplexy) OR (Cerebrovascular Stroke) OR (Cerebrovascular Strokes) OR (CVA (Cerebrovascular Accident) ) OR (CVAs (Cerebrovascular Accident) ) OR (Stroke, Acute) OR (Stroke, Cerebral) OR (Stroke, Cerebrovascular) OR (Strokes) OR (Strokes, Acute) OR (Strokes, Cerebral) OR (Strokes, Cerebrovascular) OR (Vascular Accident, Brain) OR (Vascular Accidents, Brain) OR (Acidente Vascular Cerebral) OR (Acidente Cerebral Vascular) OR (Acidente Cerebrovascular) OR (Acidente Vascular Cerebral (AVC) ) OR (Acidente Vascular Cerebral Agudo) OR (Acidente Vascular do Cérebro) OR (Acidente Vascular Encefálico) OR (Acidentes Cerebrais Vasculares) OR (Acidentes Cerebrovasculares) OR (Acidentes Vasculares Cerebrais) OR (Apoplexia) OR (Apoplexia Cerebral) OR (Apoplexia Cerebrovascular) OR (AVC) OR (AVC Agudo) OR (AVE) OR (Icto Cerebral) OR (Ictus Cerebral)) AND ((Cumplimiento y Adherencia al Tratamiento) OR (Adherencia Terapéutica) OR (Adherencia Terapéutica y Cumplimiento) OR (Adherencia al Tratamiento) OR (Adherencia y Cumplimiento Terapéutico) OR (Adherencia y Cumplimiento del Tratamiento) OR (Adhesión al Tratamiento Sometimiento al Tratamiento) OR (Treatment Adherence and Compliance) OR (Adherence, Therapeutic) OR (Adherence, Treatment) OR (Therapeutic Adherence) OR (Therapeutic Adherence and Compliance) OR (Treatment Adherence) OR (Cooperação e Adesão ao Tratamento) OR (Aderência ao Tratamento) OR (Adesão Terapêutica) OR (Adesão Terapêutica e Concordância) OR (Adesão Terapêutica e Cumprimento) OR (Adesão ao Tratamento) OR (Adesão e Concordância com o Tratamento) OR (Adesão e Conformidade com o Tratamento) OR (Adesão e Cooperação com o Tratamento) OR (Adesão e Cumprimento Terapêutico) OR (Adesão e Cumprimento do Tratamento) OR (Concordância e Adesão ao Tratamento) OR (Conformidade e Adesão ao Tratamento) OR (Cooperação e Adesão Terapêutica) OR (Cumprimento e Adesão ao Tratamento) OR (Observância e Adesão ao Tratamento) OR (Submissão ao Tratamento)) AND ((Colombia) OR (Colômbia) OR (Colombie)) AND ((Cooperación del Paciente) OR (Adherencia del cliente) OR (Adhesión del Paciente) OR (Cumplimiento del Cliente) OR (Cumplimiento del Tratamiento) OR (Cumplimiento Terapéutico) OR (Falta de Cooperación del Paciente) OR (Observancia del Paciente) OR (Paciente no Adherente) OR (Patient Compliance) OR (Adherence, Client) OR (Adherence, Patient) OR (Client Adherence) OR (Client Compliance) OR (Client Compliances) OR (Compliance, Client) OR (Compliance, Patient) OR (Compliance, Therapeutic) OR (Compliance, Treatment) OR (Compliances, Therapeutic) OR (Cooperation, Patient) OR (Non Adherent Patient) OR (Non-Adherence, Patient) OR (Non-Adherent Patient) OR (Non-Adherent Patients) OR (Non-Compliance, Patient) OR (Nonadherence, Patient) OR (Noncompliance, Patient) OR (Patient Adherence) OR (Patient Cooperation) OR (Patient Non Adherence) OR (Patient Non Compliance) OR (Patient Non-Adherence) OR (Patient Non-Compliance) OR (Patient Nonadherence) OR (Patient Noncompliance) OR (Patient, Non-Adherent) OR (Therapeutic Compliance) OR (Therapeutic Compliances) OR (Treatment Compliance) OR (Treatment Compliances) OR (Cooperação do Paciente) OR (Adesão do Cliente) OR (Adesão do Paciente) OR (Conformidade com o Tratamento) OR (Conformidade do Cliente) OR (Conformidade Terapêutica) OR (Cooperação com o Tratamento) OR (Cooperação Consciente com o Tratamento) OR (Descumprimento do Paciente) OR (Falta de Aderência do Paciente) OR (Falta de Adesão do Paciente) OR (Falta de Conformidade do Paciente) OR (Falta de Cooperação do Paciente) OR (Inconformidade do Paciente) OR (Incumprimento do Paciente) OR (Inobservância do Paciente) OR (Não Aderência do Paciente) OR (Não Adesão do Paciente) OR (Não Conformidade do Paciente) OR (Não Cooperação do Paciente) OR (Não Submissão do Paciente) OR (Observância ao Tratamento) OR (Observância do Paciente) OR (Observância do Tratamento) OR (Paciente não Aderente) OR (Cumplimiento de la Medicación) OR (Adherencia a los Medicamentos) OR (Adhesión a la Medicación) OR (Adhesión al Medicamento) OR (Adhesión al Tratamiento Farmacológico) OR (Cumplimiento de la Prescripción Medicamentosa) OR (Cumplimiento de Medicamentos) OR (No Adhesión al Tratamiento) OR (Sometimiento a la Prescripción de Medicamentos) OR (Medication Adherence) OR (Adherence, Drug) OR (Adherence, Medication) OR (Compliance, Drug) OR (Compliance, Medication) OR (Drug Adherence) OR (Drug Compliance) OR (Medication Compliance) OR (Medication Non Adherence) OR (Medication Non Compliance) OR (Medication Non-Adherence) OR (Medication Non-Compliance) OR (Medication Nonadherence) OR (Medication Noncompliance) OR (Medication Persistence) OR (Non-Adherence, Medication) OR (Non-Compliance, Medication) OR (Nonadherence, Medication) OR (Noncompliance, Medication) OR (Persistence, Medication) OR (Adesão à Medicação) OR (Aderência à Medicação) OR (Aderência ao Medicamento) OR (Aderência ao Tratamento Medicamentoso) OR (Adesão ao Medicamento) OR (Adesão ao Tratamento Farmacológico) OR (Adesão ao Tratamento Medicamentoso) OR (Cumprimento do Tratamento Medicamentoso) OR (Duração do Tratamento Medicamentoso) OR (Falta de Aderência à Medicação) OR (Falta de Adesão à Medicação) OR (Falta de Adesão ao Medicamento) OR (Não Aderência à Medicação) OR (Não Aderência ao Medicamento) OR (Não Adesão à Medicação) OR (Não Adesão ao Medicamento) OR (Persistência da Medicação) OR (Submissão ao Medicamento)) AND ((Colombia) OR (Colômbia) OR (Colombie))

**Anexo 2:** Resultados de la lista de chequeo STROBE (Strengthening the Reporting of Observational studies in Epidemiology)

Autor principal	Título del artículo	STROBE
Ospina *et al.*	Adherencia al tratamiento en pacientes diabéticos de Bucaramanga, Colombia: estudio de corte transversal	17/22
Falon *et al*.	Clínica y tratamiento de la diabetes tipo 2 en adultos jóvenes en un hospital colombiano	18/22
Rodríguez *et al.*	Prevalencia y factores asociados a la adherencia al tratamiento no farmacológico en pacientes con hipertensión arterial y diabetes en servicios de baja complejidad	19/22
Martínez- Domínguez *et al*.	Adherencia terapéutica y control metabólico en pacientes con diabetes mellitus tipo 2	16/22
Machado *et al*	Eficacia del tratamiento hipolipemiante en una muestra de pacientes de Colombia	19/22
Arciniegas *et al.*	Evaluación de costos de un programa de oxigenoterapia domiciliaria	15/22
Quiroz *et al.*	Asociación entre marcadores de posición social y adherencia al tratamiento de la hipertensión arterial en Colombia	17/22
Guarin *et al.*	Adherencia al tratamiento antihipertensivo y su relación con la calidad de vida en pacientes de dos hospitales de Bogotá, D.C. 2013-2014	19/22
Benavides *et al.*	Determinantes de adherencia al tratamiento antihipertensivo de adultos ≥ 35 años	16/22
Casas *et al.*	Adhesión al tratamiento de la hipertensión arterial en dos municipios de Colombia, 2010-2011	17/22
Castaño *et al.*	Adherencia al tratamiento de pacientes hipertensos atendidos en Assbasalud E.S.E., Manizales, Colombia, 2011	18/22
Martinez *et al.*	Frecuencia de factores de riesgo cardiovascular en pacientes hipertensos en un hospital de segundo nivel	17/22
Herrera *et al.*	Factores asociados al no control de la presión arterial en pacientes inscritos al programa de hipertensión arterial de una Entidad Promotora de Salud en Cali, Colombia, 2004	18/22
Camacho *et al*.	Self-reported prevalence of chronic non communicable diseases in relation to socioeconomic and educational factors in Colombia: A community-based study in 11 departments	18/22

**Anexo 3:** Herramientas de evaluación crítica del Instituto Joanna Briggs para revisiones sistemáticas. Lista de verificación para estudios transversales analíticos.

	Ospina	Falun	Rodríguez	Martínez-	Machado	Quiroz	Guarin	Benavides	Casas	Castaño	Martínez	Herrera
	*et al.*	*et al.*	*et al.*	**Domínguez *et al.***	*et al.*	*et al.*	*et al.*	*et al.*	*et al.*	*et al.*	*et al.*	*et al.*
Q1	Yes	Yes	Yes	Yes	Yes	Yes	Yes	Yes	Yes	Yes	Yes	Yes
Q2	Yes	Yes	Yes	Yes	Yes	Yes	Yes	Yes	No	Yes	No	Yes
Q3	Yes	N/A	N/A	Yes	Yes	Yes	Yes	Yes	Yes	Yes	Yes	Yes
Q4	No	No	No	No	No	No	No	Yes	No	No	No	Yes
Q5	Yes	No	No	No	No	No	No	Yes	No	No	No	No
Q6	Yes	No	No	No	No	No	No	No	No	No	No	No
Q7	Yes	Yes	Yes	Yes	Yes	Yes	Yes	Yes	Yes	Yes	Yes	Yes
Q8	Yes	Yes	Yes	Yes	Yes	Yes	Yes	Yes	Yes	Yes	Yes	Yes
Overall	Include	Include	Include	Include	Include	Include	Include	Include	Include	Include	Include	Include

**Anexo 4:** Herramientas de evaluación crítica del Instituto Joanna Briggs para revisiones sistemáticas. Lista de verificación para estudios de cohorte.

	**Arciniegas *et al.***	**Camacho *et al.***
Q1	Not applicable	Not applicable
Q2	Not applicable	Not applicable
Q3	Yes	Yes
Q4	No	No
Q5	No	No
Q6	Not applicable	Not applicable
Q7	Yes	No
Q8	Yes	Yes
Q9	Yes	Yes
Q10	No	No
Q11	Yes	Yes
Overall	Included	Included

**Anexo 5:** Definición de cumplimiento del tratamiento

Cuestionario	Observaciones	**Artículos**
Cuestionario Morisky- Green	Instrumento para medir el cumplimiento del tratamiento farmacológico en pacientes hipertensos mediante la respuesta a cuatro preguntas dicotómicas sobre la toma de medicamentos y las circunstancias en las que dejan de tomarlos. 1) ¿Alguna vez olvida tomar la medicación para la hipertensión arterial? 2) ¿Algunas veces olvida tomar los medicamentos a las horas indicadas? 3) Cuando se siente mejor, ¿a veces deja de tomar la medicación? y, por último, 4) Cuando se siente mal ¿a veces deja de tomar la medicación?	Ospina *et al*. Falon *et al.* Quiroz *et al*. Guarín *et al*. Benavides *et al.* Casas *et al*. Castaño *et al*. Herrera *et al.*
Cuestionario IMEVID	Instrumento autoadministrado para medir el estilo de vida en pacientes ambulatorios con diabetes mellitus tipo 2. Consta de 25 preguntas cerradas con opciones de respuesta con puntuaciones de 0, 2 y 4 -siendo 4 el valor máximo deseable- agrupadas en siete dimensiones: nutrición, actividad física, consumo de tabaco, consumo de alcohol, información sobre diabetes, manejo de emociones y cumplimiento del tratamiento.	Ospina *et al.*
Cuestionario resumen de actividades de autocuidado de la diabetes (SDSCA)	Instrumento autorreportado que evalúa el cumplimiento de las recomendaciones en los siete días previos de cinco conductas de autocuidado de la diabetes: dieta, ejercicio, automonitoreo de glucemia, cuidado de pies y tabaquismo. La escala va de 0 a 7, donde 7 es cumplimiento perfecto y 0 incumplimiento.	Martínez- Domínguez *et al.*
Cuestionario de adherencia al tratamiento para la hipertensión arterial (CAT- hipertensión arterial)	Instrumento que evalúa el cumplimiento del tratamiento farmacológico y no farmacológico en pacientes con hipertensión arterial, mediante 15 preguntas de opción múltiple. En este caso, se realizaron modificaciones a la subescala no farmacológica con adición de preguntas sobre recomendaciones específicas para diabetes, así como un ajuste en la calificación si la persona era hipertensa y diabética, o solo hipertensa. Con base en el puntaje obtenido, se categorizó como cumplidores a aquellos cuya puntuación resultó mayor o igual a 12 (rango: 5-15) y mayor o igual a 26 (rango: 8-34).	Rodríguez *et al.*
Cuestionario Martin- Bayarre-Grau	Instrumento de autorreporte que evalúa el cumplimiento del tratamiento médico indicado en pacientes hipertensos mediante 12 afirmaciones sobre su cumplimiento, implicación personal y relación transaccional (relación médicopaciente). Clasifica a los pacientes en: incumplidos (0-17 puntos), parcialmente cumplidores (18-37 puntos) y totalmente cumplidores (38-48 puntos). Las respuestas se dan en una escala Likert compuesta por cinco posibilidades de periodicidad que van desde "siempre” hasta “nunca”.	Quiroz *et al.* Castaño *et al.* Martínez *et al*
Otros	El cumplimiento terapéutico se determinó por el grado en que el paciente observó las recomendaciones consignadas por el médico en la historia clínica.	Machado *et al* Arciniegas *et al.* Camacho *et al.*
	El cumplimiento se determinó evaluando las 15 horas de utilización del oxígeno al día, calculado mediante el número de cilindros utilizados durante el mes.	
	Para evaluar el cumplimiento del tratamiento, se hizo una pregunta adicional sobre si los participantes habían estado tomando en el último mes algún medicamento en forma regular para alguna de las mencionadas enfermedades crónicas no transmisibles.	

**Anexo 6:**
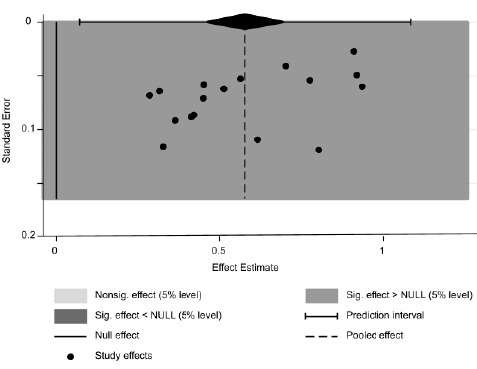
Gráfico en embudo *(funnel plot)*

**Anexo 7:**
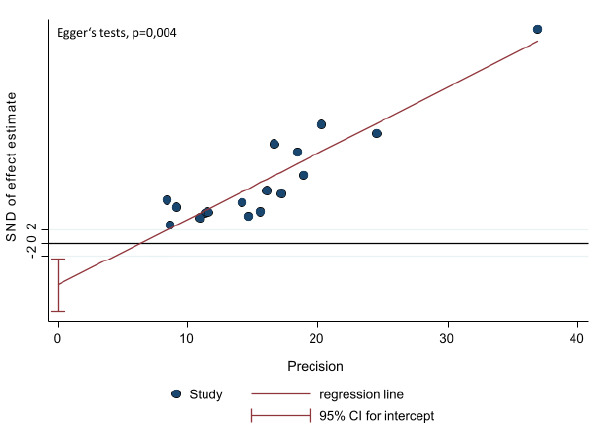
Gráfico de regresión lineal de Egger
